# Patency rates of arteriovenous fistulas created before *versus* after hemodialysis initiation

**DOI:** 10.1371/journal.pone.0211296

**Published:** 2019-01-28

**Authors:** Seonjeong Jeong, Hyunwook Kwon, Jai Won Chang, Min-Ju Kim, Khaliun Ganbold, Youngjin Han, Tae-Won Kwon, Yong-Pil Cho

**Affiliations:** 1 Division of Vascular Surgery, Department of Surgery, University of Ulsan College of Medicine, Asan Medical Center, Seoul, Republic of Korea; 2 Division of Nephrology, Department of Internal Medicine, University of Ulsan College of Medicine, Asan Medical Center, Seoul, Republic of Korea; 3 Department of Clinical Epidemiology and Biostatistics, University of Ulsan College of Medicine, Asan Medical Center, Seoul, Republic of Korea; 4 Department of Surgery, Mongolian National University of Medical Sciences, Ulaanbaatar, Mongolia; Imperial College Healthcare NHS Trust, UNITED KINGDOM

## Abstract

In an incident hemodialysis (HD) population, we aimed to investigate whether arteriovenous fistula (AVF) creation before HD initiation was associated with improved AVF patency compared with AVF creation from a central venous catheter (CVC), and also to compare patient survival between these patients. Between January 2011 and December 2013, 524 incident HD patients with identified first predialysis vascular access with an AVF (pre-HD group, n = 191) or an AVF from a CVC (on-HD group, n = 333) were included and analyzed retrospectively. The study outcome was defined as AVF patency and all-cause mortality (time to death). On Kaplan–Meier survival analysis, primary and secondary AVF patency rates did not differ significantly between the two groups (P = 0.812 and P = 0.586, respectively), although the overall survival rate was significantly higher in the pre-HD group compared with the on-HD group (P = 0.013). On multivariate analysis, well-known patient factors were associated with decreased primary (older age and diabetes mellitus [DM]) and secondary (DM and peripheral arterial occlusive disease) AVF patency, whereas use of a CVC as the initial predialysis access (hazard ratios, 1.84; 95% confidence intervals, 1.20–2.75; P = 0.005) was significantly associated with worse survival in addition to well-known patient factors (older age, diabetes mellitus, and peripheral arterial occlusive disease). Worse survival in the on-HD group was likely confounded by selection bias because of the retrospective nature of our study. Therefore, the observed lower mortality associated with AVF creation before HD initiation is not fully attributable to CVC use, but rather, affected by other patient-level prognostic factors. There were no CVC-related complications in the pre-HD group, whereas 10.2% of CVC-related complications were noted in the on-HD group. In conclusion, among incident HD patients, compared with patients who underwent creation of an AVF from a CVC, initial AVF creation showed similar primary and secondary AVF patency rates, but lower mortality risk. We also observed that an initial CVC use was an independent risk factor associated with worse survival. A fistula-first strategy might be the best option for incident HD patients who are good candidates for AVF creation.

## Introduction

Fistula-first is the general recommendation for all hemodialysis (HD) patients [1–3]. A recent meta-analysis reported that nearly two-thirds of arteriovenous fistulas (AVFs) require the use of a bridging tunneled dialysis catheter (central venous catheter [CVC]) while awaiting maturation, placing patients at increased risk of infection and that approximately 20% of AVFs are abandoned without use [4]. This increased failure rate is associated with increased vascular access (VA)-related complications and procedures [5]. Therefore, AVFs created before HD initiation seem to have improved patency and decreased abandonment compared with those created after HD initiation [4, 6, 7], and the National Kidney Foundation (NKF) recommends that AVFs be created at least 6 months before initiation of HD treatment to allow sufficient time for access creation and evaluation, vein maturation, and, if necessary, maturation-enhancing interventions before cannulation [4]. However, many of the published meta-analyses are insufficiently detailed to perform a subgroup analyses [8], and, for example, in the elderly HD population, there remains controversy as to whether the fistula-first strategy should be applied [5, 9–12], given operative risks, longer maturation times, and emerging data indicating the lack of a survival benefit compared with CVC or arteriovenous graft (AVG) use in these patients [5, 9]. Moreover, timely creation of an AVF before HD initiation is not always feasible because of the unpredictability of renal failure progression and individual variation in maturation times; premature AVF creation is associated with increased risk of VA-related complications, whereas late AVF creation cannot prevent the need for the use of a CVC.

This study aimed to investigate whether AVF creation before HD initiation was associated with improved AVF patency compared with AVF creation after placement of a CVC. We also aimed to compare other clinical outcomes, including patient survival, abandonment without use, infection, and other complications of AVF creation before *versus* after HD initiation in the incident HD population.

## Materials and methods

### Study design and population

We conducted a retrospective, observational study using data extracted from medical records. The study protocol was approved by the institutional review board of Asan Medical Center, Republic of Korea (2018–1289), which waived the requirement for informed patient consent because of the retrospective nature of our study.

Between January 1, 2011, and December 31, 2013, a total of 876 consecutive patients aged 20 years and older underwent initial upper-extremity VA creation for incident HD at our hospital, including 694 AVFs (79.2%) and 182 AVGs (20.8%). Based on previously published clinical practice guidelines [13–15], we preferred AVFs over AVGs whenever suitable vessels could be found; vessel suitability was evaluated by either physical examination alone or with additional input from duplex ultrasound findings. When forearm vessels were inadequate for the creation of a radio-cephalic fistula, we attempted to create an upper arm AVF. This study included 694 incident HD patients with confirmed initial predialysis upper-extremity AVF creation, regardless of CVC use status. A total of 112 patients with malignancies were excluded to ensure that the impact of AVF creation before HD initiation on AVF patency and patient survival, specifically, were analyzed. There were 58 additional patients who were lost to follow-up and excluded from analysis. Finally, 524 patients were included in the analysis. In our study population, all patients had a nephrologist involved in the planning of future HD and were given adequate time to arrange for VA before starting HD. Study patients were divided into a pre-HD group and an on-HD group. We identified our pre-HD group as those patients who had their first predialysis VA for AVF creation before HD initiation, and our on-HD group consisted of patients who had CVC placement for HD initiation before AVF creation without evidence of a prior AVF or AVG creation.

### Index procedures and follow-up

All VA creation procedures were performed as previously published under local anesthesia [16, 17]. In the on-HD group, a tunneled cuffed CVC was placed in the right internal jugular vein (IJV) in most right-handed patients, and in the left IJV in left-handed patients: a CVC was used for HD until the AVF was mature. In patients received a femoral venous catheter in emergency situations, CVCs were moved to the IJV within 1 week. The decision about when to attempt using a new AVF was left to the discretion of the nephrologists and HD nurses, and in the majority of cases, a CVC was removed when the AVF provided adequate HD for at least three sessions without any AVF-related complications. Postoperative surveillance was performed according to the recommendations of the clinical practice guidelines of the Society for Vascular Surgery regarding the surgical placement and maintenance of arteriovenous hemodialysis access [18].

Last follow-up data were obtained from hospital charts or follow-up physicians and by means of direct telephone interviews with the patients or their families. In this analysis, only the first event of each outcome (AVF patency and mortality) was included.

### Study outcomes and definitions

The pre-HD and on-HD groups were retrospectively analyzed and compared with regard to long-term clinical outcomes. AVF patency was the study outcome of interest; other clinical outcomes, including all-cause mortality (time to death), were also analyzed. Because the time between AVF creation and HD initiation varies, to avoid lead time bias, we measured time to outcome occurrence from the date of HD initiation. Patients who received a renal transplant after HD initiation were censored from the analysis at the time of renal transplantation. To evaluate the association between time with a CVC for HD and the occurrence of CVC-related complications, subgroup analysis according to the duration of CVC use was also performed between patients with short-term (<3 months) and long-term (≥3 months) use of CVC.

AVF maturation was defined as successful needle cannulations in addition to the achievement of prescribed HD treatment [4, 19]. A functional AVF was defined as allowing at least six adequate HD sessions without any AVF-related complications. Primary AVF patency was defined as the interval from HD initiation via a functional AVF to any intervention designed for maintenance or reestablishment of AVF function, AVF failure, or study end, whichever occurred first, and secondary AVF patency was defined as the interval from HD initiation via an AVF until the termination of HD via AVF due to any cause, regardless of the number of subsequent interventions [19]. The abandonment of AVF was defined as maturation failure or unused AVF for HD from any cause, and the duration of VA survival was the time from HD initiation via AVF or CVC to the termination of HD or study end. Maturation failure was defined as all AVFs inadequate for HD after creation. The central veins were defined as the subclavian vein, brachiocephalic vein, or superior vena cava [20]. In patients suspected of having central vein stenosis or occlusion, computed tomography, or conventional contrast venography was used to identify outflow obstruction.

### Statistical analysis

The baseline and clinical characteristics, including the VA actually used at HD initiation, the exact time of death, and the date of transplantation, as well as the clinical outcomes of the study population were tabulated according to the first predialysis VA created. Summary statistics are presented as frequencies or percentages for categorical data and means and standard deviations for continuous variables. Differences between the two groups were tested using the chi-squared test and Fisher’s exact test for categorical variables, and Student’s t-test for continuous variables. Univariate and multivariate analyses of the association of clinical variables with the study outcome were conducted with Cox proportional hazards modeling, by using the event of interest and the period from HD initiation to the date of the event or last follow-up as the outcome. Univariate Cox proportional hazard regression models were fitted to calculate hazard ratios (HRs), with 95% confidence intervals (CIs), to estimate the association of clinical variables with study outcomes. Variables with a P-value of <0.1 on univariate analysis were included in multivariate Cox proportional hazard regression models. Long-term event-free rates were estimated with Kaplan–Meier analysis and were compared with estimations calculated with the log-rank test between the pre-HD and on-HD groups. A P-value <0.05 was considered statistically significant. Statistical analyses were performed with SPSS version 21.0 (IBM Corp., Armonk, NY, USA).

## Results and discussion

The study cohort consisted of 524 incident HD patients with identified first predialysis VA creation of an AVF (pre-HD group, n = 191, 36.5%) or an AVF from a CVC (on-HD group, n = 333, 63.5%). There was no mortality or morbidity associated with AVF creation, and there were no CVC-related complications at the time of CVC placement. The baseline characteristics of the study population in relation to the initially created VA are presented in **Table 1**. There were no significant differences between the pre-HD and on-HD groups in demographics, risk factors, causes of chronic kidney disease, and type of AVF, except that patients in the pre-HD group had a higher prevalence of polycystic kidney disease than those in the on-HD group (P = 0.013). The mean follow-up duration was 45.1 months in the pre-HD group and 43.4 months in the on-HD group, with no significant difference in follow-up duration between the two groups (P = 0.461). During the study period, 33 patients died (17.3%) in the pre-HD group, and 89 died (26.7%) in the on-HD group.

**Table 1:** Baseline demographic and clinical characteristics of the study population at the onset of CKD (HD initiation) according to the initially created vascular access.

	Total	Pre-HD	On-HD	P-value
No. of patients	524	191 (36.5)	333 (63.5)	
Male	337 (64.3)	115 (60.2)	222 (66.7)	0.138
Age	55.1 ± 13.1	55.0 ± 12.8	55.1 ± 13.3	0.887
Risk factors				
DM	248 (47.3)	87 (45.5)	161 (48.3)	0.537
HTN	435 (83.0)	157 (82.2)	278 (83.5)	0.706
CVD	84 (16.0)	24 (12.6)	60 (18.0)	0.102
CVA	50 (9.5)	20 (10.5)	30 (9.0)	0.583
PAOD	14 (2.7)	2 (1.0)	12 (3.6)	0.081
Smoking	127 (24.2)	40 (20.9)	87 (26.1)	0.183
Cause of CKD				
DM	239 (45.6)	85 (44.5)	154 (46.2)	0.700
HTN	128 (24.4)	47 (24.6)	81 (24.3)	0.942
GN	62 (11.8)	23 (12.0)	39 (11.7)	0.910
PCKD	21 (4.0)	13 (6.8)	8 (2.4)	0.013
AKI	19 (3.6)	6 (3.1)	13 (3.9)	0.653
Unknown	42 (8.2)	12 (6.3)	31 (9.3)	0.224
Others	9 (1.7)	4 (2.1)	5 (1.5)	0.615
AVF				
Wrist, side to end^a^	287 (54.8)	100 (52.4)	187 (56.2)	0.400
Forearm, side to end^b^	237 (45.2)	91 (47.6)	146 (43.8)	

Continuous data are expressed as mean ± SD, and categorical data as numbers (%).

AKI, acute kidney injury; AVF, arteriovenous fistula; CKD, chronic kidney disease; CVA, cerebrovascular accident; CVC, central venous catheter; CVD, cardiovascular disease; DM, diabetes mellitus; GN, glomerulonephritis; HD, hemodialysis; HTN, hypertension; PAOD, peripheral arterial occlusive disease; PCKD, polycystic kidney disease

^a^Radio-cephalic AVF

^b^Brachio-cephalic/brachio-antecubital AVF

**Fig 1:**
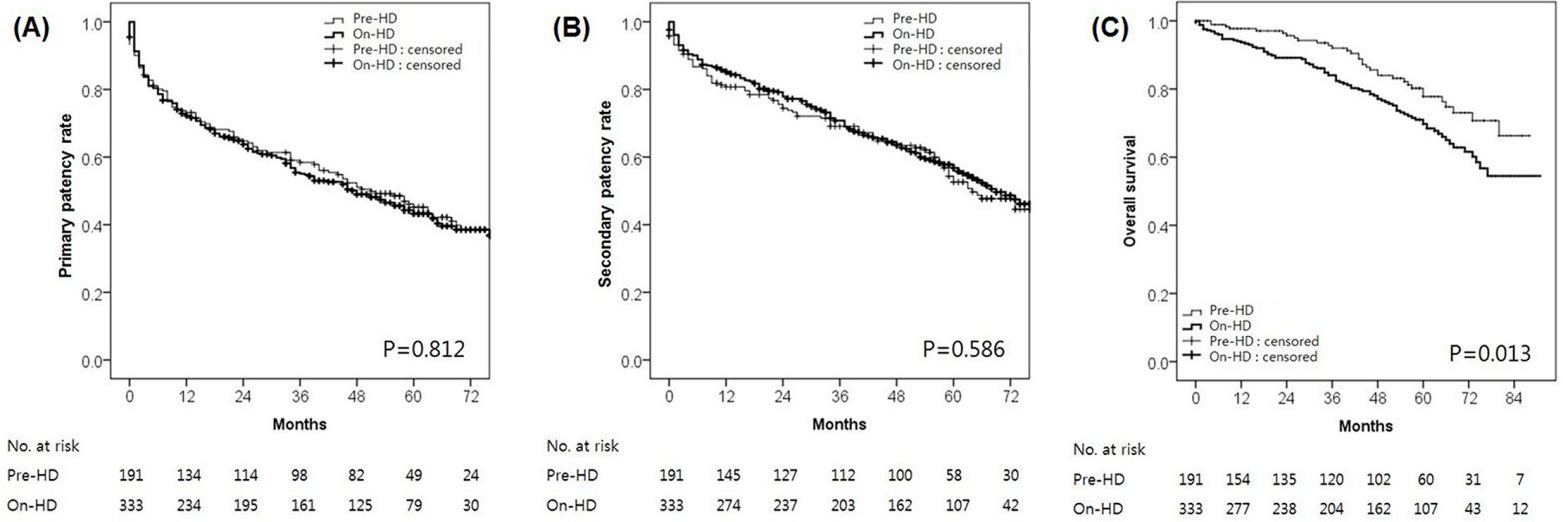
Kaplan–Meier survival analysis. Kaplan–Meier estimates of **(A)** primary and **(B)** secondary AVF patency rates, and **(C)** the overall patient survival rates in pre-HD and on-HD groups. AVF, arteriovenous fistula; HD, hemodialysis.

On Kaplan–Meier survival analysis, primary and secondary AVF patency rates did not differ significantly between the two groups (P = 0.812 and P = 0.586, respectively) (**Fig 1A and 1B**), although the overall survival rate was significantly higher in the pre-HD group compared with the on-HD group (P = 0.013) (**Fig 1C**). The median primary and secondary AVF patency durations for the pre-HD and on-HD groups were 48.8 months (95% CI, 43.6–54.0 months) and 49.0 months (95% CI, 44.9–53.2 months), and 56.6 months (95% CI, 51.6–61.5 months) and 59.7 months (95% CI, 55.9–63.6 months), respectively. The median duration of overall survival was 75.5 months (95% CI, 71.8–79.3 months) in the pre-HD group, and 70.0 months (95% CI, 66.3–73.5 months) in the on-HD group.

Clinical variables associated with outcomes were analyzed using univariate and multivariate Cox proportional hazards regression analysis. After adjustment for potential confounding variables, multivariate Cox proportional hazard regression analysis indicated that older age (HR, 1.01; 95% CI, 1.00–1.02; P = 0.006) and diabetes mellitus (DM) (HR, 1.52; 95% CI, 1.20–1.93; P = 0.001) were significantly associated with a decreased primary patency (**Table 2**); DM (HR, 1.65; 95% CI, 1.27–2.15; P<0.001) and peripheral arterial occlusive disease (PAOD) (HR, 2.27; 95% CI, 1.20–4.29; P = 0.012) were associated with secondary patency (**Table 3**); and older age (HR, 1.05; 95% CI, 1.03–1.06; P<0.001), DM (HR, 1.85; 95% CI, 1.27–2.71; P = 0.001), PAOD (HR, 2.23; 95% CI, 1.02–4.87; P = 0.045), and use of a CVC for HD initiation (HR, 1.81; 95% CI, 1.20–2.75; P = 0.005) were associated with an increased risk of mortality (**Table 4**). Long-term use of a CVC (≥3, 6, 9, 12 months, respectively), was not associated with any of study outcomes.

**Table 2:** Factors associated with primary patency in the study population.

	Univariate analysis		Multivariate analysis	
	HR (95% CI)	P-value	HR (95% CI)	P-value
Age	1.02 (1.01–1.03)	<0.001	1.01 (1.00–1.02)	0.006
Female sex	1.07 (0.84–1.36)	0.606	NA	NA
DM	1.65 (1.31–2.08)	<0.001	1.52 (1.20–1.93)	0.001
HTN	0.98 (0.72–1.34)	0.920	NA	NA
CVD	1.27 (0.94–1.72)	0.119	NA	NA
CVA	1.16 (0.80–1.69)	0.432	NA	NA
PAOD	1.69 (0.90–3.17)	0.105	NA	NA
Smoking	1.03 (0.79–1.35)	0.830	NA	NA
CVC^a^	1.22 (0.95–1.57)	0.117	NA	NA
Long-term CVC				
≥ 3 months	1.00 (0.78–1.27)	0.966	NA	NA
≥ 6 months	1.06 (0.70–1.61)	0.779	NA	NA
≥ 9 months	0.99 (0.49–1.99)	0.965	NA	NA
≥ 12 months	0.88 (0.39–1.98)	0.760	NA	NA

CI, confidence interval; CVA, cerebrovascular accident; CVC, central venous catheter; CVD, cardiovascular disease; DM, diabetes mellitus; HR, hazard ratio; HTN, hypertension; NA, not applicable; PAOD, peripheral arterial occlusive disease

^a^Use of a CVC for HD initiation

**Table 3:** Factors associated with secondary patency in the study population.

	Univariate analysis		Multivariate analysis	
	HR (95% CI)	P-value	HR (95% CI)	P-value
Age	1.01 (1.00–1.02)	0.055	1.00 (0.99–1.02)	0.521
Female sex	1.01 (0.77–1.33)	0.933	NA	NA
DM	1.68 (1.29–2.18)	<0.001	1.65 (1.27–2.15)	<0.001
HTN	0.98 (0.69–1.37)	0.889	NA	NA
CVD	1.45 (1.05–2.00)	0.025	1.24 (0.88–1.74)	0.215
CVA	1.16 (0.77–1.74)	0.482	NA	NA
PAOD	2.47 (1.31–4.65)	0.005	2.27 (1.20–4.29)	0.012
Smoking	1.11 (0.83–1.50)	0.471	NA	NA
CVC^a^	1.15 (0.87–1.52)	0.322	NA	NA
Long-term CVC				
≥ 3 months	0.98 (0.75–1.30)	0.934	NA	NA
≥ 6 months	0.98 (0.60–1.58)	0.922	NA	NA
≥ 9 months	1.04 (0.49–2.21)	0.913	NA	NA
≥ 12 months	0.87 (0.36–2.11)	0.868	NA	NA

CI, confidence interval; CVA, cerebrovascular accident; CVC, central venous catheter; CVD, cardiovascular disease; DM, diabetes mellitus; HR, hazard ratio; HTN, hypertension; NA, not applicable; PAOD, peripheral arterial occlusive disease

^a^Use of a CVC for HD initiation

**Table 4:** Factors associated with mortality in the study population.

	Univariate analysis		Multivariate analysis	
	HR (95% CI)	P-value	HR (95% CI)	P-value
Age	1.05 (1.03–1.07)	<0.001	1.05 (1.03–1.06)	<0.001
Female sex	0.92 (0.63–1.34)	0.660	NA	NA
DM	2.37 (1.63–3.45)	<0.001	1.85 (1.27–2.71)	0.001
HTN	1.19 (0.72–1.96)	0.500	NA	NA
CVD	2.30 (1.55–3.43)	<0.001	1.39 (0.92–2.10)	0.116
CVA	2.25 (1.45–3.50)	<0.001	1.40 (0.89–2.20)	0.148
PAOD	3.65 (1.70–7.84)	0.001	2.23 (1.02–4.87)	0.045
Smoking	1.32 (0.89–1.96)	0.163	NA	NA
CVC^a^	1.78 (1.18–2.68)	0.006	1.81 (1.20–2.75)	0.005
Long-term CVC				
≥ 3 months	1.10 (0.76–1.61)	0.602	NA	NA
≥ 6 months	1.16 (0.64–2.11)	0.618	NA	NA
≥ 9 months	1.03 (0.38–2.78)	0.961	NA	NA
≥ 12 months	0.65 (0.16–2.63)	0.543	NA	NA

CI, confidence interval; CVA, cerebrovascular accident; CVC, central venous catheter; CVD, cardiovascular disease; DM, diabetes mellitus; HR, hazard ratio; HTN, hypertension; NA, not applicable; PAOD, peripheral arterial occlusive disease

^a^Use of a CVC for HD initiation

Of 191 patients who had an AVF created as their first predialysis VA, 85.3% initiated HD with an AVF, whereas 14.7% of AVFs were abandoned without use because of maturation failure or other causes (**Table 5**). Among the study population, AVF abandonment occurred in 58 patients (11.1%); there was no significant difference in the maturation failure rate between the two groups (11.0% *versus* 7.5%, P = 0.175), and AVF maturation time was significantly longer in the pre-HD group compared with the on-HD group (P<0.001). According to the type of AVF, there were 30 abandonments (10.5%) in wrist AVFs and 28 (11.8%) in forearm AVFs; the primary and secondary patency rates did not differ significantly between patients with wrist and forearm AVFs (P = 0.125 and P = 0.123, respectively). Of the entire study population, 66.8% of patients initiated HD with a CVC—17 patients (8.9%) in the pre-HD group, and 333 patients (100%) in the on-HD group. According to the side of CVC, there were 13 patients using ipsilateral CVC for HD at the time of AVF creation, and four of them abandoned AVF. There were no CVC-related complications in the pre-HD group, whereas 10.2% of CVC-related complications were noted in the on-HD group. The mean duration of VA survival was similar between the two groups (45.1 ± 25.1 months *versus* 43.4 ± 25.1 months, P = 0.412).

**Table 5:** Other clinical outcomes of the study patients.

	Total	Pre-HD	On-HD	P-value
No. of patients	524	191 (36.5)	333 (63.5)	
AVF use	466 (88.9)	163 (85.3)	303 (91.0)	
AVF abandonment	58 (11.1)	28 (14.7)	30 (9.0)	0.047
Maturation failure	46 (8.8)	21 (11.0)	25 (7.5)	0.175
Transplantation^a^	2 (0.4)	2 (1.0)	0	0.061
Death (<2 months)	6 (1.1)	2 (1.0)	4 (1.2)	0.873
Other	4 (0.8)	3 (1.6)	1 (0.3)	0.108
Maturation time (months)	3.95 ± 6.0	6.93 ± 9.0	2.35 ± 2.2	<0.001
Transplantation^b^	59 (11.3)	31 (16.2)	28 (8.4)	0.009
CVC insertion^c^	350 (66.8)	17 (8.9)	333 (100)	<0.001
Ipsilateral CVC to AVF	13 (2.5)	0	13 (3.9)	
CVC-related complications^d^	34 (6.5)	0	34 (10.2)	
Duration of VA (months)	44.0 ± 25.5	45.1 ± 25.1	43.4 ± 25.1	

Continuous data are expressed as mean ± SD, and categorical data as numbers (%).

AVF, arteriovenous fistula; CVC, central venous catheter; HD, hemodialysis; VA, vascular access

^a^AVF abandonment without use due to transplantation

^b^AVF use for HD before transplantation

^c^Use of ipsilateral CVC for HD at the time of AVF creation

^d^Included CVC infection, malfunction, and symptomatic central vein stenosis

For the 350 patients who initiated HD with a CVC, we performed subgroup analysis according to the duration of CVC use: short-term (<3 months, 175 patients) and long-term (≥3 months, 175 patients) use of CVC (**Table 6**). In this subgroup analysis, we used the cutoff value of 3 months to divide short-term and long-term CVC groups because the median time with a CVC was 87 days (range, 12–632 days). The mean time with a CVC was 61.7 days in the short-term CVC group and 166.0 days in the long-term CVC group (P<0.001). Among the 175 patients with long-term CVC use, the duration of CVC use was 3–6 months in 132 patients, 6–12 months in 30 patients, and ≥12 months in 13 patients. The occurrence of CVC-related complications was significantly higher in the long-term CVC group compared with the short-term CVC group (15.4% *versus* 4.0%, P<0.001); there were increased incidences of CVC malfunction (P = 0.032) and central vein stenosis (P<0.001) in the long-term CVC group, whereas no significant between-group difference was noted in the incidence of CVC infection (P = 0.474).

**Table 6:** Baseline demographics and clinical characteristics of the patients initiated HD with a CVC according to the duration of CVC use.

	Total	Short-term CVC^a^	Long-term CVC^b^	P-value
No. of patients	350	175 (50)	175 (50)	
Male sex	228 (65.1)	117 (66.9)	111 (63.4)	0.501
Age (years)	55.0 ± 13.3	55.3 ± 13.4	54.8 ± 13.2	0.742
Time with a CVC (days)^c^	113.0 ± 89.1	61.7 ± 17.5	166.0 ± 100.7	<0.001
CVC-related complications	34 (9.7)	7 (4.0)	27 (15.4)	<0.001
Infection	8 (2.3)	5 (2.9)	3 (1.7)	0.474
Malfunction	11 (3.1)	2 (1.1)	9 (5.1)	0.032
Central vein stenosis	16 (4.6)	0	16 (9.1)^d^	<0.001

Continuous data are expressed as mean ± SD, and categorical data as numbers (%).

CVC, central venous catheter; HD, hemodialysis

^a^Use of CVC for HD < 3 months

^**b**^Use of CVC for HD ≥ 3 months

^c^Duration of CVC use for HD: median duration, 87 days, range, 12–632 days

^d^Included one CVC malfunction

Our data on incident HD patients indicated that there were no significant differences in the primary and secondary AVF patency rates between patients undergoing AVF creation before and after HD initiation. Although the worse survival observed in the on-HD group is likely confounded by selection bias secondary to the retrospective nature of our study, patients in the pre-HD group had a lower risk of death.

Although VA procedures and ensuing complications represent a major source of morbidity in HD patients [21, 22], successful creation of a functioning VA is of extreme importance to survival in this population. The three modalities of VA used for chronic HD patients are CVC, AVF, and AVG [4]. AVFs are widely recognized as the VA of first choice for most HD patients because it provides the best outcomes overall compared with AVGs or CVCs [6], related to the lower tendency for infection and thrombosis [23–25] and greater longevity [26, 27] of AVFs. CVCs have been associated with substantially higher rates of mortality, infection-related complications, central vein stenosis, hospitalization, and costs [13]; however, the use of CVCs among HD patients remains high [28], with greater than 80% of incident HD patients initiating treatment with a CVC as their primary VA according to data from US Renal Data System [29]. Consequently, the US Fistula First Breakthrough Initiative (FFBI), NKF-KDOQI (Kidney Disease Outcomes Quality Initiative), and many national guideline committees recommend AVFs as the VA of first choice for HD [8, 13–15]. Despite these recommendations, in the USA, a high proportion of patients still initiate HD using a CVC, and this proportion has not changed for nearly a decade, with only 16.9% of patients initiating HD with an AVF [30]. Moreover, recent studies have reported that creation of an AVF from a CVC had similar mortality compared with initial AVF use in elderly incident HD patients, suggesting that initial CVC use with later creation of an AVF may be an acceptable option among these patients [5, 9].

Despite substantially higher rates of CVC-related complications and the FFBI recommendations, delayed creation of an AVF from a CVC or initial AVG use is unavoidable in some patients due to a greater burden of PAOD as well as suboptimal or absent forearm and upper arm veins [31, 32]. There could be some beneficial effects of CVC use in these patients, including immediate readiness of use, relative ease of placement, and the absence of pain with cannulation [33–35], and AVGs could need to be considered as a viable option due to comparative shorter maturation times and lower rates of primary failure [9]. However, considering that the rate of decline in renal function and the time of HD initiation are often less predictable [5], in predialysis patients suitable for an AVG as the initial HD access, timely creation of an AVG without the use of CVC before HD initiation is not always feasible, and a prematurely created AVG, which may ultimately remain unused, might only cause complications, such as venous hypertension and ischemic symptoms. Therefore, in our institution, we rarely performed an AVG creation as the initial VA in predialysis patients rather than timely CVC use, and we excluded some patients who received an AVG creation before HD initiation; furthermore, given that age has not been found to be a significant determinant of AVF patency across multiple studies [36–39], we did not perform subgroup analysis according to age. We aimed to determine whether initial CVC use with subsequent creation of an AVF was associated with AVF patency and patient survival compared with initial AVF creation without CVC among incident HD patients. Compared to a prior large population-based study [5], our data showed that of incident HD patients who had an AVF as their first VA, a higher proportion of patients (85.3% *versus* 50.7%) actually used an AVF without a bridging CVC at the time of HD initiation. In addition, a fistula-first strategy was superior in terms of survival outcomes compared with a strategy of initial CVC use, although there was no significant difference in AVF patency between the two groups.

A review of AVF studies observed that nearly two-thirds of AVFs required the use of a bridging CVC while awaiting maturation and that approximately 20% of AVFs were abandoned without use [4]. Other previous studies have suggested that AVFs created before HD initiation seem to decrease abandonment compared with those created after HD initiation [4–7]. In the present study, only 8.9% of AVFs required the use of a bridging CVC in the pre-HD group; however, the abandonment rate was significantly higher in the pre-HD group than the on-HD group (14.7% *versus* 9.0%, P = 0.047), although there was no difference in the maturation failure rate between the two groups (11.0% *versus* 7.5%, P = 0.175). Furthermore, mean maturation time was significantly longer in the pre-HD group compared with the on-HD group (6.93 ± 9.0 months *versus*. 2.35 ± 2.2 months, P<0.001). Our management strategy was to attempt to create an AVF before HD initiation in the incident patients who have a slight chance for a successful AVF creation; this potentially explained the higher rate of abandonment in our pre-HD group compared with other studies [4–7]. Furthermore, a longer maturation time was noted in the pre-HD group; these patients may have had a longer waiting period before HD initiation, according to the rate of decline in renal function. We used a definition of maturation which stipulates a defined ascertainment period of successful needle cannulations in addition to achievement of the prescribed dialysis treatment [4, 19], which could have also contributed to the difference in maturation times between the two groups.

There are certain limitations to our study. First, given the retrospective and observational nature of our study, there is potential for selection and information biases on the part of physicians or patients. Hence, patients with worsening disease status, comorbidities, or older age might have been more often considered for a CVC rather than an AVF. Worse outcomes observed with CVC use are likely confounded by selection bias. Therefore, the observed lower mortality associated with AVF creation before HD initiation is not fully attributable to CVC use, but rather, affected by other patient-level prognostic factors. The incidence of CVC-related complications may have been underestimated; we did not analyze asymptomatic central vein stenosis, for example. Furthermore, the decisions to perform an AVF creation in predialysis patients were mainly made by the physician, based on the expected level of the rate of decline in renal function, vessel diameter, and vessel quality. Also, our study cohort consisted only of subjects of Asian descent; thus, our findings should be cautiously interpreted when considering other ethnic groups. Additionally, the small sample size in this single-center cohort limits the overall generalizability of our results. Finally, as with all observational studies, our study does not confirm a causal relationship between an AVF creation before HD initiation and survival outcomes. Additional large cohort studies are required to establish the association between the fistula-first strategy and the clinical outcomes of these incident HD patients.

In conclusion, among incident HD patients, compared with patients who underwent creation of an AVF from a CVC, initial AVF creation showed similar primary and secondary AVF patency rates, but lower mortality risk. Despite aforementioned limitations, a fistula-first strategy might be the best option for incident HD patients who are good candidates for AVF creation. Future studies are needed to determine the underlying mechanisms, and to develop individualized VA management strategies among incident HD patients.

## Supporting information

S1 Data Data of 524 incident HD patients with identified first predialysis VA placement of an AVF or of an AVF from a CVC. (XLSX)
